# Research on Cement-Free Grouting Material for Shield Tunneling in Water-Rich Karst Regions

**DOI:** 10.3390/ma18061192

**Published:** 2025-03-07

**Authors:** Zheng Che, Tian-Liang Wang, Zheng-Guo Zhou, Shuo Wang, Xin-Wei Ma

**Affiliations:** 1 China Construction Sixth Engineering Bureau Hydropower Construction Co., Ltd., Tianjin 300222, China; chezheng0314@163.com (Z.C.); wangtianliang2024@163.com (T.-L.W.); zzg111121@163.com (Z.-G.Z.); 2 China Construction Sixth Engineering Bureau Co., Ltd., Tianjin 300450, China; 3 State Key Laboratory of Simulation and Regulation of Water Cycle in River Basin, China Institute of Water Resources and Hydropower Research, Beijing 100038, China; 4 School of Ocean Engineering, Harbin Institute of Technology, Weihai 264209, China

**Keywords:** sustainable materials, double-liquid grouting, bentonite, viscosity

## Abstract

With the increasing number of anti-seepage reinforcement projects and the continuous improvement of quality requirements, high-performance and green requirements have also been put forward for grouting materials. Traditional karst cave grouting mainly uses cement-based grouting materials, which not only have high carbon emissions but also do not comply with the sustainable development strategy with regard to being green, low-carbon, and environmentally friendly. A green grouting material made by mixing a slurry A and slurry B is proposed in this paper. The solid phase of slurry A is composed of stone powder and bentonite, for which an anti-washout admixture is necessary. Slurry B is a suspension of thickener (CMC or HPMC) and anhydrous ethanol. By mixing the two slurries evenly, the grouting material is obtained. Experiments were used to investigate the ideal ratios of stone powder, bentonite, and water in slurry A, and the ratio of thickener to anhydrous ethanol in slurry B, and to analyze the development and evolution of the apparent viscosity of slurry A and slurry B after mixing. This study revealed that the optimum ratio of stone powder and bentonite was 4:1, and the most reasonable water–solid ratio was 0.8:1.0. The optimum ratio of anhydrous ethanol to CMC or HPMC in slurry B was 5:1. Slurry B was added to slurry A at a rate of 5~10% to obtain the best grouting material properties. The proposed mixed grouting material would not disperse even in flowing water and could harden and consolidate quickly. The strength of the consolidation grouting body was close to that of wet soil, which can meet requirements for tunnel construction.

## 1. Introduction

In recent decades, subway construction has provided a powerful impetus for rapid economic development, especially in China. As of the beginning of 2024, there are a total of 48 cities in China with subways, including 41 in mainland China and 7 in Hong Kong, Macau, and Taiwan, with a total mileage of 80,185 km. Almost all of the cities with subways are expanding their subway sizes [1,2]. In subway transportation construction, shield tunneling is commonly used because of its high efficiency and safety [3,4]. Due to Karst strata being widely distributed in China, water-rich rock karst caves frequently become hydro-geological hazards in the process of tunnel excavation [5,6]. Grouting plays a crucial role during shield tunneling. Many construction accidents, such as surface subsidence, segment drift, and adverse loading conditions of segmental rings, are associated with improper grouting [7]. Grouting methods are widely used in karst treatment however, because of the advantages of their simple operation and their good treatment effects [8].

Grouting, widely used in civil engineering, is a method of pouring suitable slurries that can be condensed into a cracked water-bearing rock layer, concrete, or a loose soil layer, to reduce in situ permeability and improve strength, in order to achieve reinforcement, seepage resistance, and waterproofing goals [9,10]. With advancements in science and technology, the use of grouting materials has opened up more areas of use, and, of course, higher performance requirements have been put forward [11,12,13]. Grouting materials injected into karst caves need to harden and consolidate in a relatively short period to significantly improve the conditions of the surrounding strata and contribute to safer tunneling construction conditions [14]. Grouts are commonly a suspension that can naturally hydrate and harden, including cementitious materials such as lime or cement, along with fly ash, sand, cement or lime, water, and chemical additives, including superplasticizers, dispersion resistance agents, setting accelerators, retarder agents, early strength agents, water-retaining agents, micro-expansive agents and viscosity modifying admixtures (VMAs) [15,16,17]. Cement-based grouts and lime-based grouts have been successfully used in some cases in tunnel engineering for karst treatment [18].

However, the traditional grouts used for shield tunnels have performance defects. Cement-based grouts have excellent impermeability, compressive strength and flexural strength in terms of body hardening. But the fluidity of this grouting material is relatively poor, and grouting pipes are easily blocked due to the large particle size and lower inject-ability of grout. It is difficult to meet the different requirements of various geological formations [19,20,21]. Lime-based grouts have excellent fluidity and low bleeding rates. However, lime increases volume shrinkage and reduces the compressive strength of hardened bodies, especially in the case of quicklime [22,23]. The diverse applications of grouts in shield tunnels demonstrate the limited adaptability of these grouts [11,24]. Meanwhile, achieving optimal performance in terms of both the fresh-state properties and the mechanical properties of the hardened body, as well as scour resistance and durability, is challenging. Furthermore, some grouts’ properties may even conflict with each other when trying to meet specific constituent material ratios simultaneously [25,26].

Regarding the performance requirements of grouts for shield tunnels, grouts should have good fluidity and little segregation for long-distance transportation, and have characteristics that are not easily diluted by groundwater. When a grout fills a space evenly, the early strength the grout is relatively uniform, and its numerical value will be equal to that of intact soil [27,28,29]. It is clear that the strength requirement for the grouted body is not significantly higher. Therefore, according to the above requirement, clay and bentonite can be used as raw materials for new grouting materials. Bentonite is composed, mainly, of a hydrous magnesium–calcium aluminum silicate called montmorillonite, which is a clay mineral of the smectite group, and it is a natural mineral, non-toxic and harmless [30,31]. Bentonite can expand 10–30 times when it comes into contact with water, forming a dense gel structure, effectively filling cracks and sealing leakage channels, and is especially suitable for leak-stopping projects in dynamic water environments [32,33]. Meanwhile, utilizing the technology of double liquid grouts, the bentonite and stone powder constitute slurry A, while the thickening and hardening agent constitutes slurry B. The performance of the grouts undergoes a significant transformation upon mixing slurry A and slurry B. This rapid mixing prevents material segregation and ensures that the grouts are not diluted by groundwater prior to the initial setting [34,35].

Therefore, in this paper, a low-cost and environmentally friendly cement-free grouting material for shield tunnels in water-rich karst areas was proposed. A series of tests were conducted to investigate the formulation, optimum ratio, rheological properties, and mechanical properties of the proposed grouting material after consolidation and hardening. It was hoped that this research would provide a sustainable and effective grouting reinforcement formulation, extending the engineering application of bentonite as a grouting material.

## 2. Materials and Methods

### 2.1. Materials

The grouting material proposed in this paper was a two-component grouting material, the components of which were named slurry A and slurry B, respectively. Slurry A was the main component and slurry B was the thickening and hardening agent. Components A and B were prepared, respectively. The two components were mixed together evenly at the construction site before they were pumped into the karst cave.

The main mineral of bentonite is montmorillonite, the theoretical chemical formula of which is Al_2_[Si_4_O_10_](OH)_2_·nH_2_O. The bentonite used in this study was an earthy yellow, delicate powder. Particles of less than 10 microns accounted for more than 50%. The particle size distribution was shown in Table 1.

The stone powder used in this project was generated during the processing of stone aggregates and the mixing of concrete mixes, and was collected by dust collection equipment. Its main components, measured by XRF, were calcium carbonate (85%), silicon dioxide (12%), and other clay mineral components. The results of the particle size analysis of the stone powder were shown in Table 2. The stone powder was relatively coarse, with a median particle size (D50) of 20.03 microns. To ensure the cohesion of the proposed grouting material under flowing water, anionic polyacrylamide with a molecular weight of 8 to 10 million was used in slurry A as an anti-dispersant.

**Figure 1:**
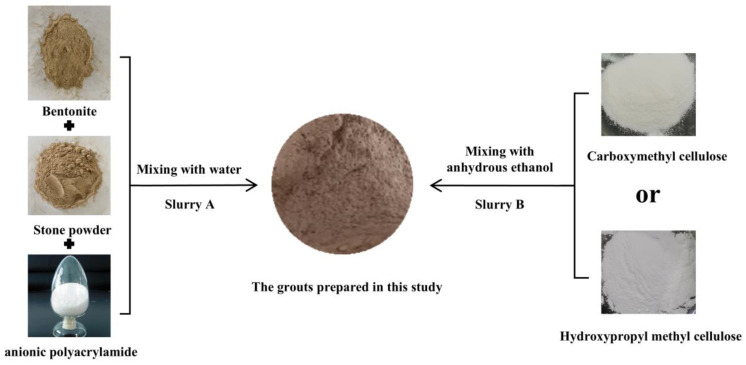
Schematic diagram of the grout’s formation process.

Carboxymethyl cellulose (CMC) and hydroxypropyl methyl cellulose (HPMC) were used as thickening agents in this study to increase the viscosity of the proposed grouting material, which can consolidate and harden in a relatively short time. The solubility of the thickener in water was very small, so anhydrous ethanol was used as a carrier for the thickener. When the demand for slurry A was high, a large amount of water was required to dissolve all the required thickener. After mixing slurry A and B, the thickening time was prolonged, and the strength of the hardened grout body was obviously affected due to the high water content. Therefore, in this research, anhydrous ethanol and thickener were mixed to make a suspension, namely slurry B, and sufficient thickener was added to the slurry without increasing the water content. The mass fraction of ethanol was 99.7%. A schematic diagram of the grout’s preparation process was shown in Figure 1.

### 2.2. Experimental Methods

Slurry A was made of bentonite, stone powder, and an anti-dispersion agent, and was evenly mixed with water, while slurry B was a suspension made of a thickening agent and anhydrous ethanol. The NDJ-99 viscometer (Shanghai Precision Instrument Co., Ltd., Shanghai, China) was used to measure the viscosity of slurry A, according to the standard GBT 43876-2024 (test method for viscosity of cement paste, standardization administration of China) [36]. The viscosity requirement is based on the permeability (<10^−5^ cm/s) of the rock layer, and the apparent viscosity of the slurry (500~10,000 mpa·s achieves the grouting requirement [22]. When the rotor rotates in the slurry, it is subject to viscosity resistance. The higher the viscosity of the slurry, the greater the viscous resistance moment acting on the rotor. The viscous resistance moment acting on the rotor was detected by the sensor, and the viscosity and critical yield value of the slurry were obtained. The technical index of the viscometer was shown in Table 3.

Slurry B consisted of the appropriate amount of anhydrous ethanol mixed with a certain amount of thickening agent. After the optimally proportioned slurry A and slurry B were thoroughly stirred and mixed in different ratios, the changes in rheological properties and the development of mechanical strength of the grouted materials were established by physical observation and tests.

## 3. Results and Discussion

### 3.1. Slurry A and Its Performance

**Figure 2:**
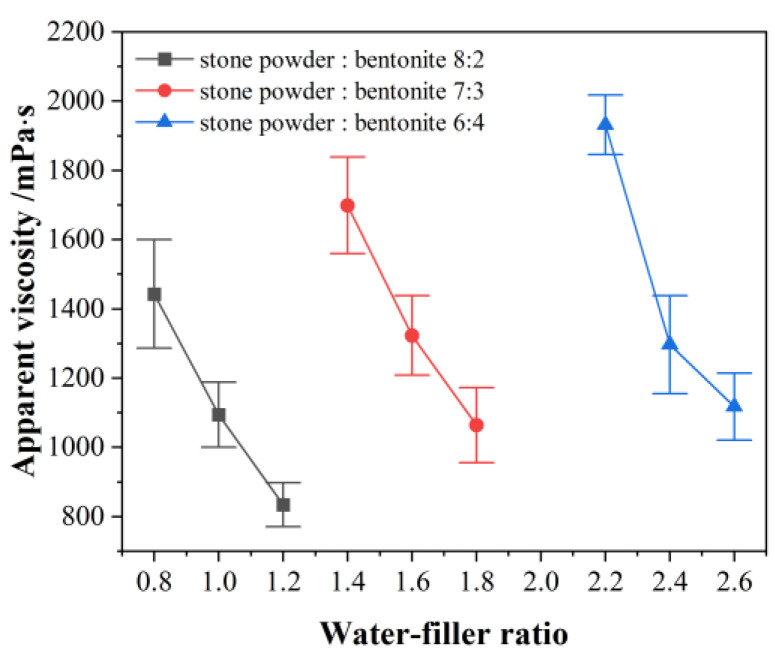
Change in apparent viscosity of slurry A with different water–filler ratios.

Slurry A was obtained by mixing filler (bentonite and stone powder), and anti-dispersant, together with water, in the mixer. The preliminary test showed that when the bentonite content in slurry A was high, the water demand was large and the slurry was thick, which was not conducive to it being pumped. To ensure the fluidity of slurry A and facilitate pumping, the ratio of stone powder to bentonite in slurry A was determined to be between 3:2 and 4:1. The mixing proportions of slurry A were shown in Table 4. The change in apparent viscosity with the water–filler ratio was plotted in Figure 2.

For the three different compositions, the apparent viscosity of the slurry decreased with an increase in the water–filler ratio. The increase in the amount of bentonite led to a significant increase in water demand, and the water–filler ratio increased correspondingly. The apparent viscosity of the slurry increased with increases in the proportion of bentonite. The viscosity of the grout was based on the requirements for injecting rock formations [22]. The viscosity of lime-based grouting material was on the same order of magnitude as the grout’s viscosity in this study, but the difference was that as the lime content increased, the viscosity of the slurry decreased [37]. Analysis of variance (ANOVA) for the water–filler ratio and stone powder–bentonite ratio are shown in Table 5. ANOVA showed that water–filler ratio and stone powder–bentonite ratio had a statistically significant influence at a level of 5% (*p* lower than 0.05) on apparent viscosity.

**Figure 3:**
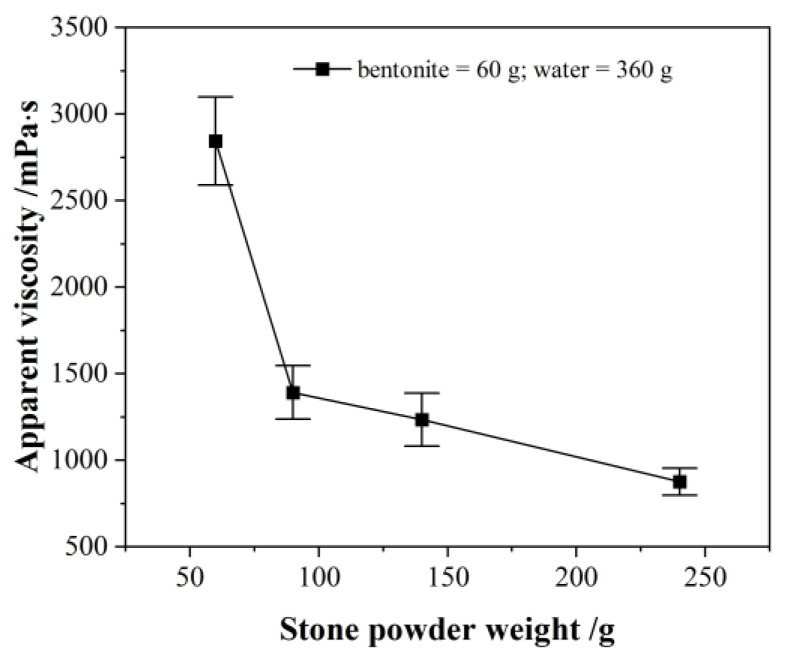
Effect of stone powder on apparent viscosity.

When the weight of bentonite and water remain unchanged, only the weight of the stone powder increased, and the viscosity of the slurry continued to decline within a certain range, as shown in Figure 3. The ANOVA data are shown in Table 5. It can be seen that reducing the amount of bentonite, and increasing the amount of stone powder, appropriately, could reduce water consumption. This helped to shorten the thickening time, and improved the strength of the slurry after hardening. In terms of the thickening time of the slurry A, the optimum bentonite quantity was taken to be 20% to 30% of the filler.

### 3.2. Slurry B and Its Performance

The effective ingredient in slurry B was a thickening agent, and its main function was to promote the rapid thickening of the mixed slurry after mixing with slurry A. In this research, Carboxymethyl cellulose (CMC) and hydroxypropyl methyl cellulose (HPMC) were used as thickeners. These two thickeners had less solubility in water and could form a viscous liquid after being dissolved in water. To add a sufficient amount of thickener to the slurry A requires large amounts of water as a carrier. However, mixing in a large amount of water was not conducive to the thickening of the mixed slurry and or maintaining high strength in the hardened grouting material. The thickener was insoluble in ethanol; therefore, anhydrous ethanol was used as the carrier and mixed with the thickener to form a suspension as the slurry B. The optimum mass ratio between the thickener and anhydrous ethanol was 1:5.

**Figure 4:**
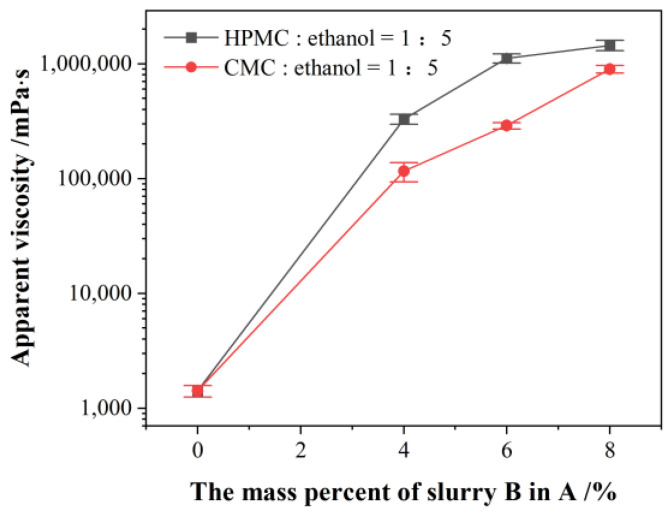
Effect of slurry B on apparent viscosity.

Mixing stone powder and bentonite in a mass ratio of 4:1, slurry A was formed according to a water–filler ratio of 0.9. The relationship between the viscosity of the mixed slurry and the amount of slurry B is shown in Figure 4. It reveals that the apparent viscosity of the mixed slurry rapidly increased by 2~3 orders of magnitude within a few minutes after slurry A and slurry B are uniformly mixed in a certain proportion. When the addition of slurry B reaches or exceeds 10% of slurry A, the mixed slurry loses its plasticity completely. As can be seen from Figure 3, when slurry B was mixed with slurry A and its optimum proportion was 5~10%, the apparent viscosity of the proposed grout material was large, and the apparent viscosity was not ideal if the proportion was too large or too small.

Once the grouting material was poured into the karst cave, consolidated, and hardened, the threat of groundwater compression to shield tunneling construction was eliminated. Although the grout body had a certain strength after drying, the strength of the grout body was close to the strength of the wet soil. It must be admitted that the hardening strength of this grouting material is far less than that of cement-based grouting material, which is its defect. There was no practical significance in the study of its strength. The next step will be to conduct on-site tests in a practical tunnel project to verify the long-term stability of this grouting material under dynamic water pressure stress.

## 4. Conclusions

Based on double-liquid grout technology, a low-cost and environmentally friendly cement-free grouting material for shield tunnels was proposed. The performance of the grouts was studied with viscosity tests. According to the above experimental results, the following conclusions are obtained:

1. The optimal mass ratio of stone powder to bentonite in slurry A was 4:1 (with a water–solid ratio of 0.8:1.0), and the ratio of thickening agents (CMC or HPMC) to anhydrous ethanol in slurry B was 1:5. At this ratio, the slurry had good fluidity. Meanwhile, the viscosity increased rapidly by 2–3 orders of magnitude with the addition of 5–10% slurry B. Therefore, the grout could harden rapidly.
2. With the increase in bentonite content, the slurry viscosity increased, and the best proportion of bentonite in grouting was found to be 20~30%, to avoid huge losses in fluidity. With increases in stone powder content, the slurry viscosity decreased, and the stone powder could increase the fluidity of the grouts. The stone powder–bentonite-based grouts proposed in this study significantly reduced the carbon emissions of traditional cement-based materials.
3. Using anhydrous ethanol as a carrier, more thickening agents could be admixed without entrapping a large amount of water. This helps to thicken the cement-free grouting material quickly, while maintaining a high plastic strength.

## Figures and Tables

**Table 1:** Particle size distribution of bentonite.

Particle Size (μm)	0–1	1–5	5–10	10–20	20–45	45–75	75–100
Content (%)	5.51	36.97	12.54	13.03	13.28	10.00	4.93

**Table 2:** Particle size distribution of stone powder.

Particle Size (μm)	0–5	5–10	10–20	20–45	45–75	75–100	100–300
Content (%)	10.10	10.08	19.60	15.06	13.55	6.77	13.97

**Table 3:** The technical index of the viscometer.

Type	Measuring Range/mPa·s	Rotor Diameter/mm	Rotor Speed/rpm
NDJ-99	20~2 × 10^6^	18, 15, 10, 3	0.3, 0.6, 1.5, 3, 6, 12, 30, 60

**Table 4:** Mixing proportions of slurry A.

Test ID	Stone Powder/%(*w*/*w*)	Bentonite/%(*w*/*w*)	Water–Filler Ratio	Anti-Dispersant Content /%
1	80	20	0.8	0.8
2	80	20	1.0	0.8
3	80	20	1.2	0.8
4	70	30	1.4	0.7
5	70	30	1.6	0.7
6	70	30	1.8	0.7
7	60	40	2.2	0.6
8	60	40	2.4	0.6
9	60	40	2.6	0.6

**Table 5:** Analysis of variance (ANOVA) for the water–filler ratio, stone powder–bentonite ratio, stone powder weight, and the mass percentage of slurry B in A.

Variance	Sum of Squares (SS)	Degree of Freedom	Mean Square (MS)	*F*	*p*
Water–filler ratio	679,744.044	2	339,872.022	3.314	<0.05
Stone powder–bentonite ratio	3,707,598.376	8	463,449.797	35.103	<0.05
Stone powder weight	6,752,774.789	3	2,250,924.93	76.127	<0.05
The mass percentage of slurry B in A	4,900,327,163,786.12	3	1,633,442,387,928.7	19.956	<0.05

## Data Availability

The original contributions presented in this study are included in the article. Further inquiries can be directed to the corresponding authors.
